# Pharmacogenomics-Guided Antidepressant Therapy as a Solution to Treatment-Resistant Depression: A Systematic Review

**DOI:** 10.1192/bjo.2025.10191

**Published:** 2025-06-20

**Authors:** Leah Rose Alexander

**Affiliations:** University of Buckingham Medical School, Buckingham, United Kingdom

## Abstract

**Aims:** Treatment-resistant depression (TRD), defined as failure to achieve remission despite adequate antidepressant trials, remains a pervasive clinical conundrum, linked to reduced quality of life, increased mortality, and higher healthcare costs. Pharmacogenomics-guided treatment (PGT) offers a potential solution to the ongoing challenge of TRD by using genomic testing to identify genetic factors influencing drug metabolism and response. This systematic review aimed to assess whether PGT improves symptom remission in adults with TRD compared with treatment as usual (TAU). Secondary objectives included evaluating the impact of PGT on treatment response and overall symptom improvement.

**Methods:** A systematic review of clinical studies published up to 8 July 2023 was conducted across PubMed and Cochrane Library databases based on Preferred Reporting Items for Systematic Reviews and Meta-Analyses (PRISMA) guidelines. Search terms were defined using the ‘Population, Intervention, Comparison, Outcome’ (PICO) framework. Studies were selected using predefined inclusion and exclusion criteria. Clinical trials or observational studies evaluating the efficacy of PGT for TRD in at least 10 adult participants were included. The quality of the included studies was assessed using the Critical Appraisal Skills Program (CASP) checklist.

**Results:** The systematic literature search identified 307 records, 15 of which were included in a qualitative synthesis. Among 14 studies evaluating remission, 13 (92.9%) reported improvements with PGT compared with TAU, with 8 (57.1%) showing significant gains. For treatment response, 12 out of 13 studies (92.3%) demonstrated enhancements with PGT, 8 (61.5%) of which were significant. All 8 studies assessing symptom improvement reported benefits with PGT, with 5 (62.5%) showing significant results. Notable findings included more consistent effects in patients with fewer prior antidepressant failures and improved response rates even in the absence of remission. However, variability in study designs, scoring systems, and definitions of TRD limited the generalisability of results.

**Conclusion:** PGT demonstrates promise in enhancing remission, treatment response, and symptom outcomes in TRD. By tailoring treatment to individual genetic profiles, this approach may reduce the trial-and-error process associated with antidepressant therapies, thereby improving patient well-being and alleviating healthcare burdens. Nevertheless, further studies, including meta-analyses and cost-benefit evaluations, are warranted to establish the clinical and economic viability of PGT for widespread implementation in healthcare systems like the NHS.

